# Successful Treatment of Mirizzi’s Syndrome Using SpyGlass Guided Laser Lithotripsy

**DOI:** 10.4021/gr447w

**Published:** 2012-07-20

**Authors:** Hussain Issa, Bahaa Bseiso, Fadel Almousa, Ahmed H. Al-Salem

**Affiliations:** a Department of Internal Medicine, King Fahad Specialist Hospital, Dammam, Saudi Arabia and Department of Pediatric Surgery, Maternity and Children Hospital, Dammam, Saudi Arabia

**Keywords:** SpyGlass, Laser lithotripsy, Mirrizi’s syndrome, Retained cystic duct stone

## Abstract

The majority of common bile duct stones can be effectively treated by endoscopic sphincterotomy and stone extraction using basket or balloon extractor. Stones more than 2 cm in diameter on the other hand require mechanical, electrohyraulic lithotripsy and sphincterotomy and balloon dilation. Mechanical lithotripsy may not be successful because of the size, consistency and site of the stones. In these cases, laser lithotripsy is the treatment of choice. This however requires direct visualization of the stone which may not be feasible for impacted cystic duct stones. This report describes the successful treatment of difficult cystic duct stones in two patients with Mirizzi’s syndrome type I using per oral Spyglass and intraductal holmium: YAG Laser Lithotripter.

## Introduction

Mirrizi’s syndrome is a rare cause of obstructive jaundice that is caused by extrinsic compression of the common hepatic duct usually from a stone impacted in Hartmann’s pouch or cystic duct [1]. A standardized open surgical approach was advocated for patients with Mirizzi’s syndrome but with the recent advances in minimal invasive surgery, laparoscopic approach was also advocated but this is still controversial and should be done only by experienced laparoscopic surgeons [2-5]. In the era of minimal invasive surgery and in order to avoid bile ducts injuries, subtotal cholecystectomy and/or leaving a long cystic duct has gained popularity [5-7]. This is more so in difficult and complicated cases such as Mirrizi’s syndrome which puts them at risk of developing post-cholecystectomy syndrome. A retained cystic duct stone is seen in 16% of patients with post-cholecystectomy syndrome which poses diagnostic and therapeutic difficulties. Several modalities of treatment were suggested to treat retained cystic duct stones [8, 9]. Surgery, open or laparoscopic was suggested but these are not without risk considering the scarring and adhesions from the previous surgery [9, 10]. Other less invasive alternative techniques have been tried. These include ERCP with an extraction basket, mechanical, electrohydraulic or laser lithotripter [11-15]. These modalities of treatment are more valuable for retained, difficult common bile duct stones as they are more accessible. The problem with retained cystic duct stones such as those in Mirrizi’s syndrome is they are not easily accessible to such treatment. This report describes the successful treatment of difficult, cystic duct stones in two patients with Mirrizi’s syndrome type 1 using per oral Spyglass and intraductal Laser Lithotripter.

## Case Report

### Case 1

**Figure 1:**
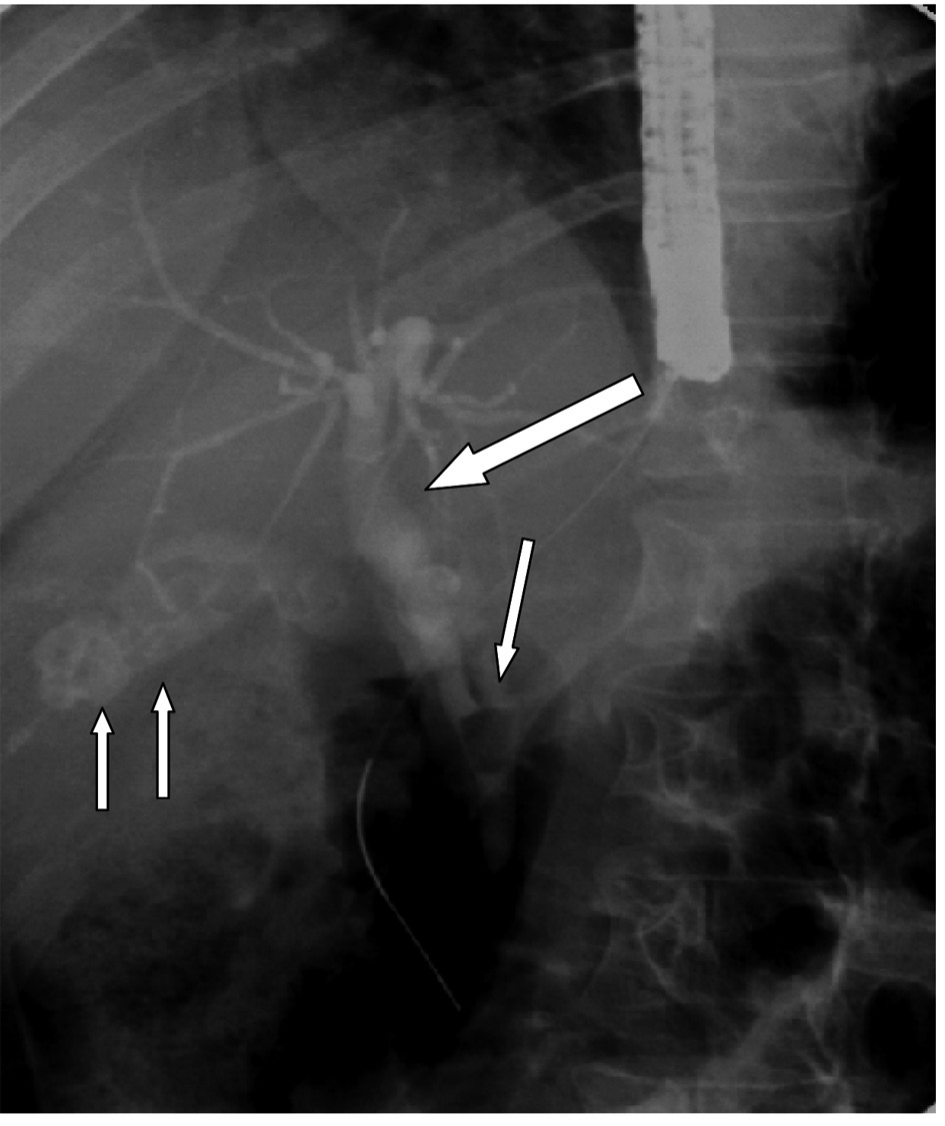
ERCP showing gallstones (two arrows), a long cystic duct and cystic duct stone causing Mirrizi’s syndrome (single arrow). Note the dilated common bile duct proximally (thick arrow).

**Figure 2:**
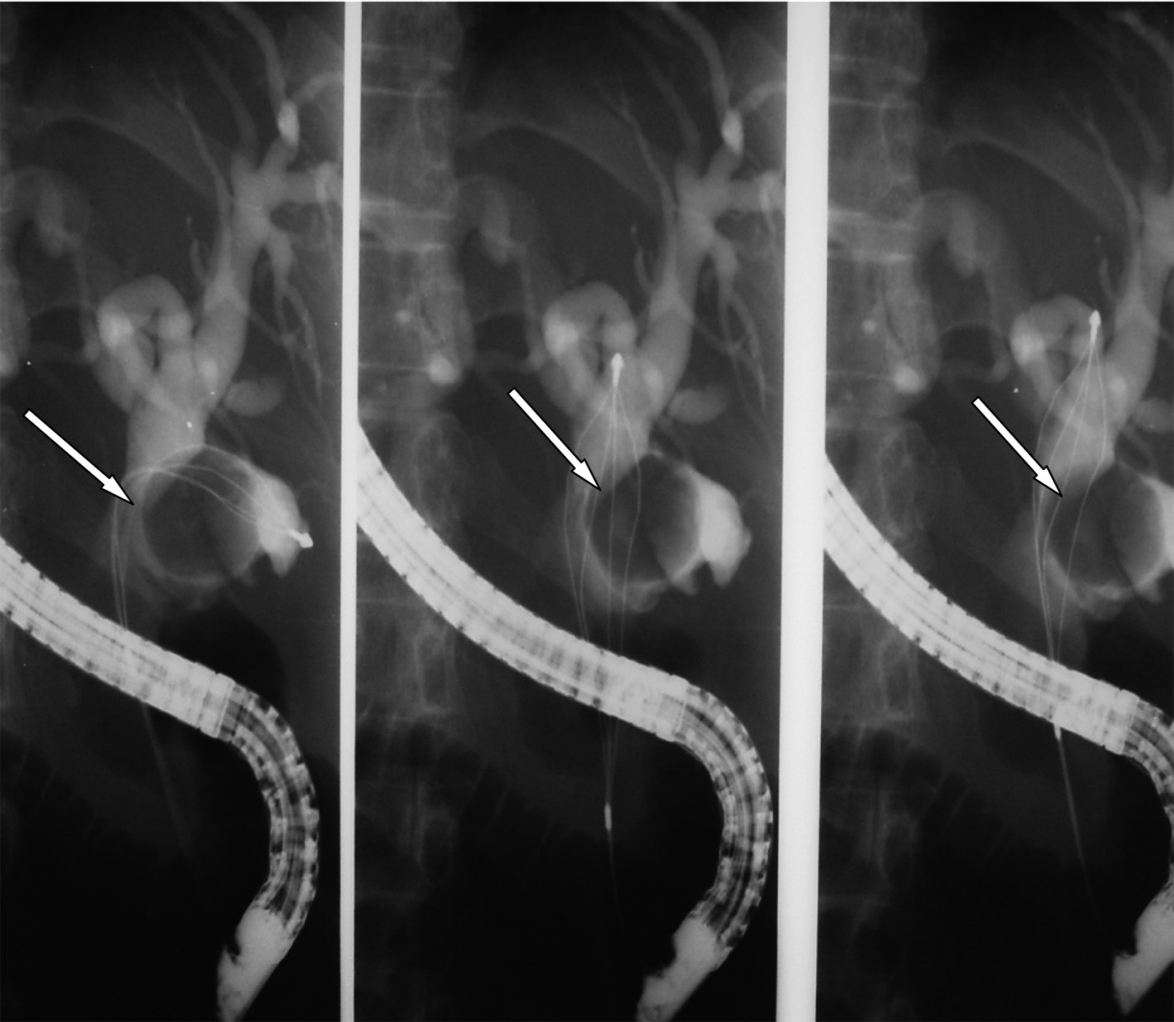
ERCP showing the remnant cystic duct with a stone impacted in it causing Mirrizi’s syndrome (arrow).

**Figure 3:**
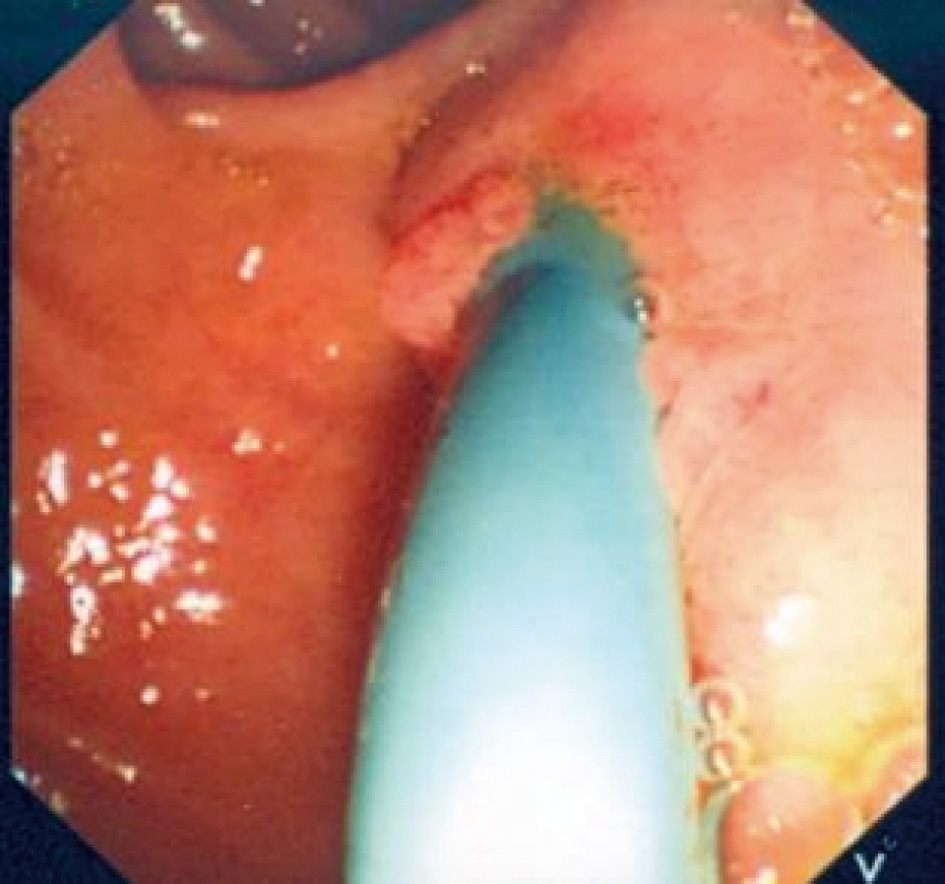
A photograph showing the SpyGlass passing through the ampulla of Vater.

**Figure 4:**
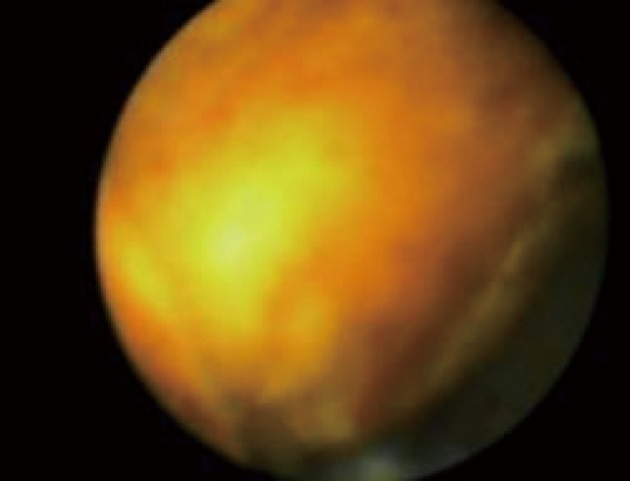
Impacted cystic duct stone viewed via the Spyglass cholangioscope.

**Figure 5:**
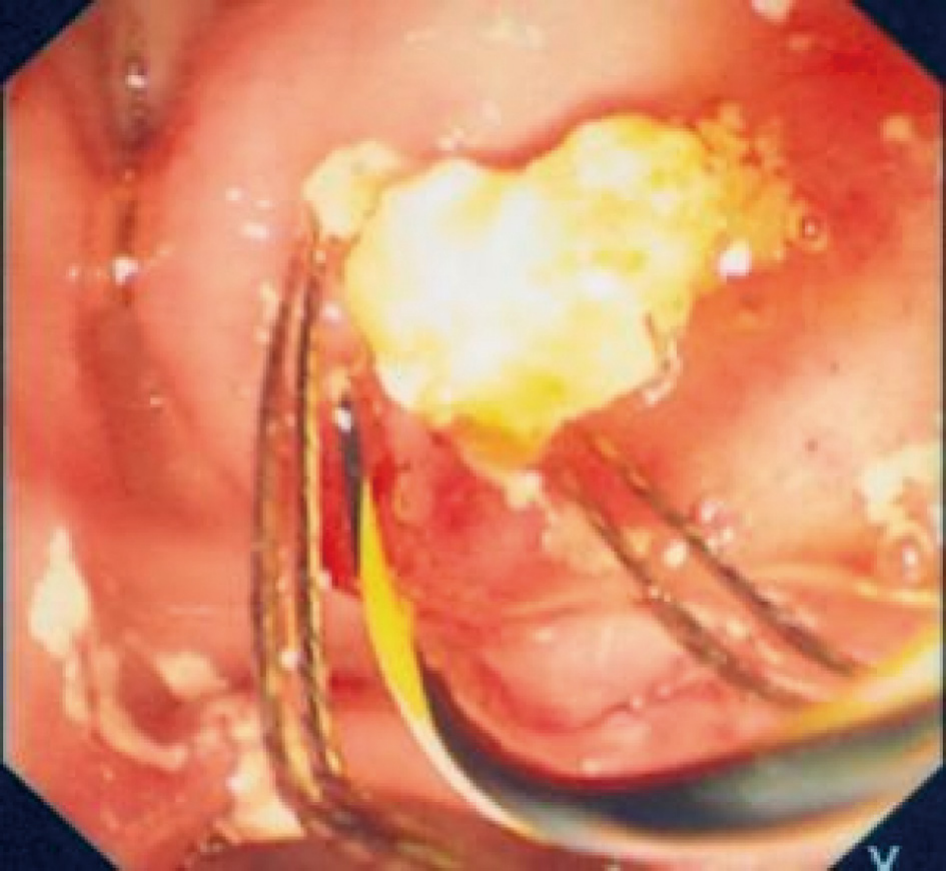
Fragmented stone removed by a basket and emergining from the ampulla of Vater.

**Figure 6:**
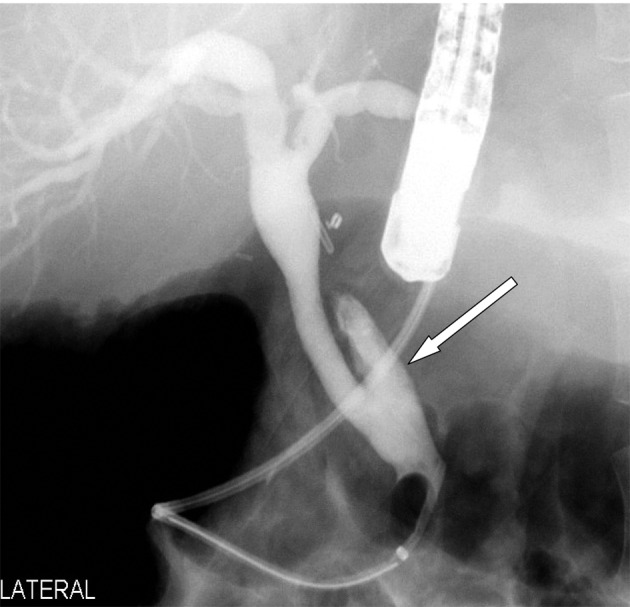
ERCP showing the remnant cystic duct after its clearance by the Spyglass cholangioscope (arrow).

A 25-year-old female was found to have obstructive jaundice secondary to choledocholithiasis and was referred to our hospital for ERCP. This showed Mirizzi's syndrome type 1 causing an obstructive jaundice (Fig. 1). A biliary stent was inserted and we recommended open cholecystectomy. However, when she went back to her referring hospital, she underwent laparoscopic cholecystectomy. Few days post surgery, her obstructive jaundice was not relieved and she was sent back for ERCP and stent removal. Upon doing the 2nd ERCP, she was noticed to have three stones impacted in the cystic duct at junction with the common bile duct consistent with Mirizzi’s syndrome type 1. Two subsequent ERCP’S Attempts to extract the stones by two experienced gastroenterologists and with different manipulations were unsuccessful. She underwent open resection of the remaining part of the gallbladder and Hartmann pouch with stone extraction from cystic duct. One month postoperatively, she presented for a follow-up ERCP and stent removal. The ERCP showed dilated CBD and common hepatic duct and a remaining stone in the cystic duct (Fig. 2). Several attempts to extract this stone using trapezoid basket were unsuccessful due to the size and shape of the stone and its impaction into the cystic duct. A 10-French, 10 cm stent was inserted and the plan was to arrange for an ERCP plus Spyglass and laser lithotripsy. This was performed (ERCP, Spyglass and intraductal laser lithotripter) in which the following was seen: Initially attempts to extract the stone in the cystic duct using trapezoid basket were not successful. The Spyglass was inserted (Fig. 3) and cholangioscope and cystic ductoscopy showed a large yellowish stone in the cystic duct (Fig. 4). There were no stones in the CBD. Using the holmium laser calculase device, the probe with frequency 6, energy 1.2 - 1.7 was used and the light directed over the stone. Pulses were initiated several times resulting in fragmentation of the stone. These stone fragments were extracted (Fig. 5) and the cholangiogram confirmed that CBD and Cystic ducts were stones free (Fig. 6). After that, the patient’s jaundice gradually resolved and on follow-up six months later, she was doing well with no complaints.

### Case 2

**Figure 7:**
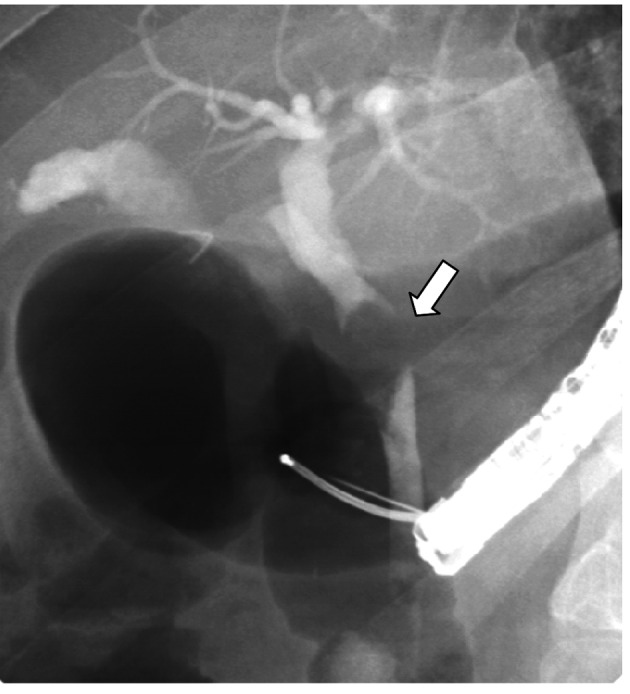
ERCP showing an impacted large cystic duct stone causing Mirrizi’s syndrome.

**Figure 8:**
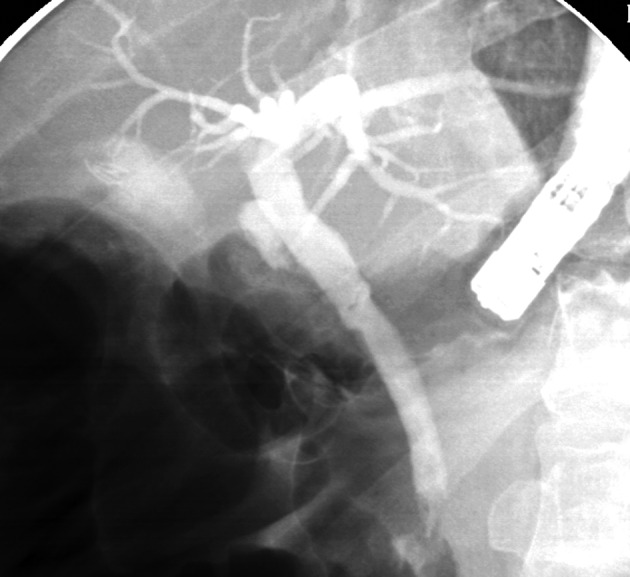
ERCP showing clearance of the cystic duct via the Spyglass cholangioscope.

A 53-year-old male patient, was a known case of chronic hepatitis C and controlled hypertention. He presented complaining of right upper quadrant abdominal pain of 5 days duration, radiating to the back and not associated with nausea, vomiting, jaundice or diarrhea. Laboratory workup showed total bilirubin = 226 µmol/liter, direct bilirubin = 195 µmole/liter, ALP = 243 unit/liter, AST = 105 unit/liter, ALT = 167 unit/liter, GGT = 422 unit/liter and albumin = 4 g/L. CBC, Renal/ electrolytes and coagulation profile were normal. Abdominal ultrasound showed dilated CBD with 15 mm stone. ERCP was performed twice and showed impacted cystic duct stone with dilated duct due to extrinsic compression of the stone consistant with Mirizzi’s syndrome (Fig. 7). Attempts for stone extraction were unsuccessful in the two occasions and a biliary stent was inserted. He was then planned for intraductal laser lithotripsy. SpyGlass cholangioscope was inserted, a large yellow stone was visualized impacted at the cystic duct insertion, laser was pointed to the stone and in close contact with stone, Holmium laser 1.7 jole and frequency of 6 pulses/second was discharged and complete stone fragmentation was achieved. The stone fragments were then extracted (Fig. 8).

## Discussion

Cholelithiasis is one of the common conditions and Laparoscopic cholecystectomy is now the treatment of choice. To obviate the risk of bile duct injuries, several modifications were made including subtotal cholecystectomy and leaving a long cystic duct which was reported in 16% of patients with post-cholecystectomy syndrome [7-9]. The remnant cystic duct calculus is one of the causes of post-cholecystectomy syndrome. This is seen more in patients with Mirizzi’s syndrome where difficulties may arise because of the impacted stone at the cystic duct or Hartmann’s pouch. Mirizzi’s syndrome is an unusual complication of cholelithiasis and occurs in approximately 0.05-2.7% of all patients with gallstones [2]. The syndrome was first described in 1948 by Pablo Luis Mirizzi and is characterized by impaction of stones in the cystic duct or neck of the gallbladder (Hartmann’s pouch), compressing and resulting in mechanical obstruction of the common hepatic or common bile duct leading to intermittent or persistant jaundice [1]. McSherry in 1982 suggested a sub classification of the Mirizzi’s syndrome into two types. In Type I there is external compression of the common hepatic or common bile duct by a calculus in the cystic duct or Hartmann's pouch whereas, in type II the stone erode partially or completely into the common hepatic duct resulting in a cholecysto-choledochal fistula [4]. In 1989, a new classification of Mirizzi’s syndrome was published [16]. Type I lesions are those with external compression of the common bile duct; in type II lesions a cholecystobiliary fistula is present with erosion of less than one third of the circumference of the bile duct; in type III lesions, the fistula involves up to two thirds of the duct circumference; and in type IV there is complete destruction of the bile duct. Open surgery is the treatment of choice for patients with Mirizzi’s syndrome. The role of laparoscopy in the treatment of Mirizzi’s syndrome remains controversial. This is even so in type I Mirizzi’s syndrome where laparoscopic cholecystectomy is not always feasible considering the inflammatory tissue in the area of Calot's triangle which offers a high operative risk during dissection. Others advocate laparoscopic surgery in the treatment of Mirizzi’s syndrome but to obviate bile duct injuries and retained cystic duct stones this must be done by experienced laparoscopic surgeons [2, 3, 5].

Retained common bile duct stones are not rare and considering the local post-operative scarring of the area, surgery should be avoided as this is associated with significant postoperative morbidity and there are several more feasible modalities of treatment. ERCP, endoscopic sphincterotomy and common bile duct stones extraction is the treatment of choice and it is successful in 85-90% of patients using conventional balloons and baskets [17, 18]. This however is not successful in those with stones > 2 cm in diameter who usually require mechanical lithotripsy, sphincterotomy and balloon dilatation, electrohydraulic lithotripsy, or laser lithotripsy prior to stone extraction. Mechanical lithotripsy is an excellent method, easy to perform, inexpensive and usually effective in 80-90% of cases. Failures with this technique result from inability to entrap the stone in the lithotripsy basket. This is usually because of the size of the stone or its location. In our patients this method was not going to be successful not only because of the size of the stone but more important because of the location of the stone in the cystic duct which makes it inaccessible. Failure to do mechanical lithotripsy will necessitates either electrohydraulic or laser lithotripsy. This however requires direct visual control. Electrohydraulic lithotripsy is rarely used because of its high potential for tissue damage and bleeding. In our patients, the stones were impacted in the cystic duct, which means that direct visual control using the ordinary per oral cholangioscope and a smaller, more maneuverable mother-baby-scope system is needed. This requires the presence of two gastroenterologists [19]. In our patients, this was achieved using the single-operator per oral SpyGlass cholangioscope and intraductal laser lithotripter. SpyGlass requires just one physician operator, and provides direct visualization of all bile-ducts. This direct visualization with the SpyGlass enables a single physician to definitively diagnose and perform therapeutic intervention in one procedure. SpyGlass procedures proved to be clinically feasible not only in term of clinical diagnosis but also providing adequate samples for histological diagnosis, and successfully guided electrohydraulic and laser bile duct stone lithotripsy [20-24]. This is more so for difficult bile duct stones. Electrohydraulic lithotripsy was used successfully with SpyGlass to treat bile duct stones [20, 23]. To the best of our knowledge, these are the first two cases in which the SpyGlass and laser lithotripsy was successfully used to treat difficult cystic duct stones in patients with Mirrizi’s syndrome type I. The SpyGlass proved to be clinically feasible, provided adequate visualization and successfully guided laser lithotripsy for difficult cystic duct stones. The procedure was safe and well tolerated.

In conclusion, retained bile duct stones are not rare and there are several techniques to extract them depending on the size, site and consistency. Retained cystic duct stones on the other hand are less common and because of their location they are not easily accessible endoscopically. This is more so for those with large stones causing Mirrizi’s syndrome. We found the single-operator per oral SpyGlass cholangioscope and intraductal laser lithotripter a useful and effective technique to manage these stones. Early utilization of this technique will spare patients from repeated ERCP with its associated complications. Such technique should however be used in specialized centers and by experienced gastroenterologists.
